# Physiotherapy Rehabilitation in an Infected Non-union Shaft of Femur Repair Patient: A Case Report

**DOI:** 10.7759/cureus.50786

**Published:** 2023-12-19

**Authors:** Anushri R Patil, Deepali S Patil, Medhavi V Jagzape

**Affiliations:** 1 Musculoskeletal Physiotherapy, Ravi Nair Physiotherapy College, Datta Meghe Institute of Higher Education and Research, Wardha, IND

**Keywords:** strengthening, orthopaedic, trauma, barthel index, osteomyelitis, infection, rehabilitation, femur shaft fracture, non-union

## Abstract

While definitions may vary, infected non-union is generally described as a condition where a fracture fails to heal due to infection, typically persisting for a duration of six to eight months. Infected non-unions occurring in the shaft of the femur are infrequent and typically result from severe open fractures with deep fragmentation and segmental bone loss or following internal fixation of a severely fragmented closed fracture. Some associated factors contributing to non-union include positive bacterial cultures from deep wounds, histological evidence of bone necrosis, exposed bone without a vascularized periosteum for more than six weeks, and the presence of purulent discharge. Osteomyelitis, stiffness in adjacent joints, smoking, loss of soft tissue resulting in multiple sinus tracts, osteopenia, and deformities leading to limb length discrepancies are all complicating factors that impact treatment and prognosis. Infected non-union of bones, although rare, presents a significant challenge for physiotherapists striving to provide appropriate treatment. The level of stabilization at the fracture site is the most critical factor influencing whether a fracture progresses to non-union or successfully heals. Infection, such as osteomyelitis, also contributes to the development of non-union. Additionally, issues like tissue atrophy, joint stiffness, and muscle contractures can further complicate the non-union of a bone, posing a considerable challenge for physical therapists in helping patients achieve their recovery goals. Top of form this case report reviews the case of a 35-year-old male who was reported to Acharya Vinoba Bhave Rural Hospital (AVBRH) with an infective non-union of the shaft of the femur fracture after two months of repair. This case report highlights the recovery of patients from post-operative complications like non-union, stiffness, and reduced range of motion through tailored physiotherapy rehabilitation and improved quality of life.

## Introduction

The hip constitutes a synovial joint of the ball-and-socket variety, linking the pelvic girdle to the lower limb. This joint involves the fusion of the femoral head with the acetabulum of the hip bone (femur). Pelvic fractures often result from significant trauma, typically arising from low-impact falls in older women and young men [1-3]. Diagnosing a non-union fracture is typically a complicated process, with factors generally falling into three categories: mechanical, biological, and infection-related. Infection is observable in approximately 40% of non-union fractures, particularly in cases of impaired wound healing or open fractures. Interestingly, in 5% of all non-union cases, infection is present without any discernible clinical symptoms or serological indicators [4,5]. Factors that increase the likelihood of delayed healing and non-healing include patient-related aspects like older age, underlying medical conditions, smoking, the use of non-steroidal anti-inflammatory drugs, different genetic disorders, metabolic diseases, and nutritional deficiencies [6-8]. A total of 14 patients suffering from infected non-union of the humerus complicated by sinus discharge underwent a staged treatment protocol comprising two steps: comprehensive debridement along with the implantation of local antibiotic beads and external skeletal fixation combined with autologous bone grafting. During the initial stage, a thorough debridement and removal of sequestrum were performed. Antibiotic beads were utilized to fill the bone defect, and the wound was subsequently closed directly. In the second stage, the antibiotic bead chains were replaced with cancellous bone grafts from the patient's own body. At the same time, unilateral Hoffman external skeletal fixators were applied. The average follow-up period was 73.6 months (ranging from 29 months to 9 years). The time taken to achieve bone union varied between 3.5 and 8 months (with an average of 4.3 months). There were two cases with complications related to the Hoffman pins, which were then converted to internal plate fixation. All infections were successfully eliminated, and wound healing occurred without the need for additional skin grafts or flap coverage. With the exception of one patient who unfortunately passed away, all fractures achieved bone union. Following the removal of external fixation and the implementation of physical therapy, most patients regained satisfactory elbow motion and preserved joint function. The two-stage approach proved to be effective for managing infected humeral non-union, not only eradicating infection and achieving bone union but also preserving limb function and joint mobility [9].

According to a population-based analysis, the overall risk of non-unionization per fracture is 1.9%, rising to 9% in the peak age group of 25-44 years [10,11]. Despite improvements in the design of implants and our understanding of biomechanics, addressing non-union in femoral shaft fractures remains a formidable task when treating such injuries. Dealing with femoral non-union poses a substantial treatment challenge for healthcare providers, leading to significant personal and financial burdens for the affected patients [12,13]. Despite advancements in surgical procedures, alternatives for fracture fixation, and healing adjuncts, femoral non-union remains a great clinical issue [14,15]. Due to the seriousness of the injury, initial fixation that may be insufficient, and patient demographic factors such as age, medical conditions, and nicotine use, femoral fractures can sometimes fail to heal properly. The femur is surrounded by three well-defined muscular compartments. The anterior or extensor compartment contains the femoral nerve, which aids in knee extension. The posterior or flexor compartment houses the sciatic nerve, responsible for knee flexion. The medial compartment accommodates the adductor muscles, and within it, the obturator nerve can be found. Surrounding and attaching to the proximal femur and shaft are the gluteal muscles, which include the gluteus medius, minimus, and maximus and are associated with the inferior and superior gluteal nerves. Depending on the location of the fracture, these muscles in femoral shaft fractures exert forces that can cause deformity in the fractured segments. The proximal segment is generally affected by flexion, abduction, and external rotation by the iliopsoas and hip abductors. Meanwhile, the quadriceps and hamstrings tend to pull the distal segment in a proximal direction (shortening it), and the adductor muscles move it toward adduction [16]. There are various treatments available, like the Ilizarov fixator, plating, and intramedullary nailing fixation using open reduction internal fixation through non-union [17,18]. Physiotherapy also plays a key role in rehabilitating the patient and making their recovery in a safe and quicker manner [19].

## Case presentation

Patient information

In this case, we have a 35-year-old male farmer who presented to Acharya Vinoba Bhave Rural Hospital (AVBRH) with complaints of pus discharge over the lateral aspect of his right thigh for the past two days. The patient was apparently alright till December 25, 2022, when, while riding his motorbike with his friend, he sustained a road traffic accident (RTA) and a collision with a two-wheeler near his hometown at around 11:00 am, sustaining an injury to his right thigh. Immediately after the fall, he developed pain and deformity in his right thigh, which was so excruciating that he was unable to stand on his leg. The pain was severe in intensity, provoked by movements, and relieved by immobilization. It was also associated with bony deformity and swelling. He was taken to the Government Medical College, where he was diagnosed with a mid-shaft femur fracture on the right side and was managed with interlock nailing on the right side on December 27, 2022, under spinal anesthesia. There was no history of loss of consciousness, no history of seizures, no history of ear, nose, or throat (ENT) bleeding, and no history of nausea or vomiting. The patient came to AVBRH with the above complaints on March 5, 2023. The patient is complaining of pus discharge over the lateral aspect of the mid-thigh's (suture line) right side. Later, he was further managed and was referred for physiotherapy.

Clinical findings

**Figure 1:**
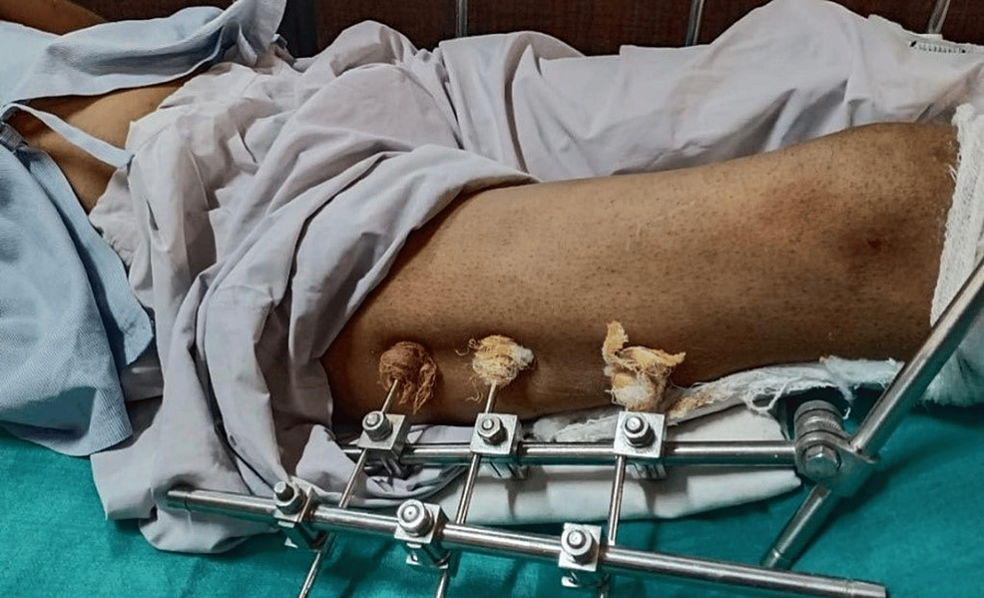
Post-operative stage

The patient was examined at the same level, with bilateral hips in the spine position. Upon inspection and observation, the patient found it hard to do all the movements on the affected side while there was a normal range of motion on the unaffected side. The overlaying skin was red. A suture line was present, swelling was seen, pus discharge was seen, and there was no sinus or dilated vein. Bony tenderness was elicited; there was a local rise in temperature; no obvious bony deformity was seen. Active ankle movements were seen and the popliteal artery was palpable. The manual muscle testing (MMT) and range of motion (ROM) (lower limb) taken pre-operatively are mentioned in Tables 1-2, respectively. Figure 1 shows the patient's post-operated limb with an external fixator. Table 3 represents the timeline of events.

**Table 1:** Manual muscle testing (pre-operative) N/A: not assessable

Muscle	Left side	Right side
Hip joint		
Flexion	3	N/A
Extension	3	N/A
Abduction	3	N/A
Adduction	3	N/A
Knee joint		
Flexion	3	3
Extension	3	3
Ankle joint		
Plantarflexion	3	3
Dorsiflexion	3	3

**Table 2:** Range of motion (pre-operative)

Joint	Left side	Right side
Hip joint		
Flexion	0–120°	0–80°
Extension	2–0°	15–0°
Abduction	0–40°	0–25°
Adduction	0–30°	0–20°
Knee joint		
Flexion	0–125°	0–90°
Extension	125°–0°	90–0°
Ankle joint		
Plantarflexion	0–40°	0–40°
Dorsiflexion	0–10°	0–10°

**Table 3:** Timeline of events

Timeline	Date
Date and time of accident	December 25, 2022
First operation (open reduction and internal fixation) for shaft of femur fracture	December 27, 2022
The patient reported to hospital with complaints of pus discharge and swelling	March 05, 2023
2nd operation: non-union fixation with external fixator	March 08, 2023

 Diagnostic investigation

**Figure 2:**
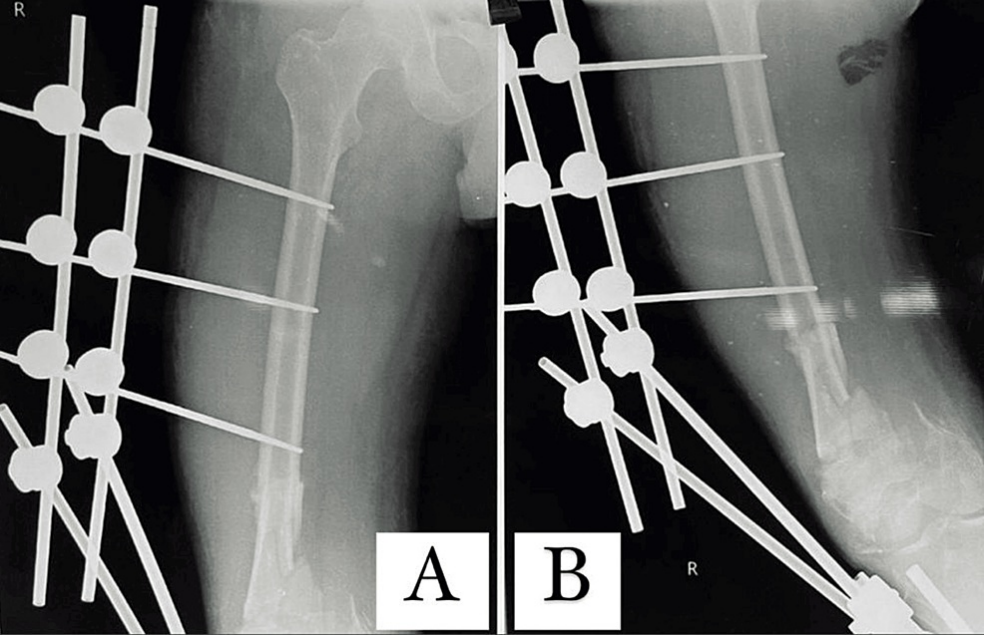
X-ray of non-union of shaft of femur is evident (A) Anterior view; (B) lateral view In anterior view and lateral view of X-ray, external fixator is seen attached to shaft of femur

 X-ray shown in Figure 2 shows evident non-union of shaft of femur.

Therapeutic management

The patient received rehabilitation for three weeks. Table 4 represents the physiotherapy treatment protocol given to the patient.

**Table 4:** Therapeutic intervention ADLs: activities of daily living, ROM: range of motion, PROM: passive range of motion

Phase	Goals	Therapeutic exercise
Phase I: Immediate postoperative phase (week 1–2)	Precaution	No active ROM of hip joint, avoid prone and side lying on the affected side, avoid ADLs, no ambulation
	To decrease inflammation	Application of cryotherapy for 10–15 minutes
	To reduce tenderness and pain	Thermotherapy, 2 times a day; ice pack application for 8–10 minutes
	To maintain range of motion and functional activities	PROM exercise for hip flexion and extension, isotonic exercises (ankle toe movements), upper limb mobility exercise with help of weights (1-liter water bottle), shoulder shrugs
Phase II: week 2–6	To reduce inflammation	Cryotherapy for 10–15 min
	To increase ROM	Active assisted ROM exercises for hip flexion and extension initiated in this phase, isometric exercise for quadriceps and hamstring muscle (10 repetition with 5 sec hold)
	To improve strength and endurance and functional activities	Closed chain exercises for hip (10 repetition, 3 sets), strengthening of uninvolved extremities, toe touching weight bearing (room ambulation), upper limb mobility exercises continued
Phase III: intermediate phase (6–8 week)	To increase ROM and muscle strength, endurance and functional activities	Active ROM of hip flexion, extension, adduction, abduction and external, internal rotation (10 repetition, 3 sets), isometrics exercises to knee and ankle (10 repetition, 3 sets), partial weight bearing with walker was started in this phase (10 reps, 2 times), upper limb mobility exercises – crunches (10 repetitions, 2 sets)
Phase IV: 8–12 weeks	To improve ROM and muscle strength and endurance and functional activities	Active ROM of hip flexion, extension, adduction, abduction and external, internal rotation (12 repetition, 3 sets), isometric exercises to knee and ankle, upper limb mobility exercise- crunches (10 repetition, 3 sets) continued partial weight bearing with walkers (hall ambulation)
	Home program	Dynamic isometrics quadriceps (10 repetitions, 3 set), upper limb mobility exercise using water bottle, wand. Hall ambulation 5 rounds.

Follow up

Table 5 represents outcome measures. Post-operative ROM is mentioned in Table 6 and MMT is mentioned in Table 7.

**Table 5:** Outcome measures

Outcome measure	Pre-physiotherapy intervention (post-op day 1)	Post-physiotherapy intervention
Numerical pain rating scale	8/10 on activity 5/10 on rest	5/10 on activity 3/10 on rest
Lower-extremity functional scale	30	65
Barthel’s index	40	85

**Table 6:** Range of motion (post-operative)

Joint	Left side	Right side
Hip joint		
Flexion	0–120°	0–30°
Extension	0–20°	0–5°
Abduction	0–40°	0–10°
Adduction	0–30°	0–10°
Knee joint		
Flexion	0–125°	0–70°
Extension	125–0°	70–0°
Ankle joint		
Plantarflexion	0–40°	0–35°
Dorsiflexion	0–10°	0–10°

**Table 7:** Manual muscle testing (post-operative)

Muscle	Left side
Hip joint	
Flexion	3
Extension	3
Abduction	3
Adduction	3
Knee joint	
Flexion	3
Extension	3
Ankle joint	
Plantarflexion	3
Dorsiflexion	3

Due to the fixator, the patient's range of motion on the right side is reduced. Manual muscle testing of the right side is not mentioned due to not being assessable due it to the fixator.

## Discussion

In this case report, a 35-year-old male with a non-union femur shaft fracture is discussed and managed by open reduction. By addressing the specific needs and limitations of the patient, the rehabilitation process effectively promotes healing, restores function, and improves overall well-being. The prime goal is to avoid subordinate issues, improve the ROM and strength of hip muscles, and become functionally independent as soon as possible. Post-op rehabilitation begins with close collaboration between the surgeon, medical staff, the patient, and the rehabilitation therapy team following non-union fixation. Nevertheless, it is essential to acknowledge the individual nature of such cases, as factors like age, comorbidities, and overall health status may influence the rehabilitation protocol [19]. Isometric quadriceps exercises help maintain muscle tone and prevent atrophy in the context of an infected non-union without putting undue strain on the healing site. This is especially important in the early stages of rehabilitation to help with overall knee stability. It is critical for a comprehensive rehabilitation approach to progress from isometric exercises to open and closed-chain exercises. Closed-chain exercises in particular aid in replicating weight-bearing situations, gradually restoring functional strength and improving joint proprioception [20]. Gait training is a necessary and important component of rehabilitation. It is started in two to four weeks, with toe-to-toe weight bearing first, then partial weight bearing, and finally full weight bearing. An assessment of the surgical findings is necessary to establish rehabilitation guidelines. Outcome measures that are used, like the numerical pain rating scale, the lower extremity functional scale, and Barthel's index, were found to be improved after three weeks of rehabilitation. This communication continues throughout the rehabilitation procedure and is crucial to successful results. The goal is to reduce stress on the restored tissues and bones in order to facilitate early tissue and bone healing. The average procedure begins with a passive range of motion on the first post-op day while preserving tolerable pain levels and then begins gait training for quicker functional independence.

## Conclusions

Infective non-union is a common complication of a lower limb fracture. Early detection plays a part in management. Treatment options involve open repair or closed repair. Physical therapy plays an important part in the rehabilitation of non-union femur shaft fractures. This case report emphasizes the critical role of physiotherapy rehabilitation in managing patients with infected non-union of the shaft of the femur repair. The successful outcome of the presented case demonstrates the efficacy of an organized and customized physiotherapy program in facilitating optimal recovery and functional restoration. The patient's mobility and muscle strength improved as a result of a combination of targeted exercises, range of motion techniques, and progressive weight-bearing activities, resulting in an improved overall quality of life. This study illustrates the importance of including physiotherapy as part of the treatment plan for patients with infected non-union of femoral shaft fractures, and it provides useful insights for future clinical management in similar cases.
